# Supplementary material for: Relative Contribution of Metal Content and Soil Particle Mass to Health Risk of Chromium-Contaminated Soil
Source: Int J Environ Res Public Health. 2022 Apr 26;19(9):5253. doi: 10.3390/ijerph19095253 (PMC9100977; doi:10.3390/ijerph19095253)
Supplement: Supplementary file 1 [file ijerph-19-05253-s001.zip › ijerph-1671721-supplementary.pdf]

International Journal of
Environmental Research and Public Health

Table S1. Correlation matrices among the sample properties and the Cr bioaccessibility.

Soil properties	Particle size	OM (mg·kg⁻¹)	Fe (mg·kg⁻¹)	pH	Cr(mg·kg⁻¹)	CEC (cmol⁺·kg⁻¹)	PBET-G	PBET-GI	SBRC-G	SBRC-GI
Low										
Particle size	1	-0.285	-0.928**	0.377	-0.949**	-0.512	0.670**	0.848**	0.789**	0.04
OM (mg·kg⁻¹)		1	0.329	-0.305	0.227	0.191	-0.111	-0.069	-0.006	0.176
Fe (mg·kg⁻¹)			1	-0.43	0.894**	0.407	-0.630*	-0.733**	-0.636*	0.138
pH				1	-0.2	-0.072	0.01	0.124	0.048	-0.123
Cr(mg·kg⁻¹)					1	0.609*	-0.829**	-0.912**	-0.846**	-0.156
CEC (cmol⁺·kg⁻¹)						1	-0.778**	-0.718**	-0.746**	-0.657**
PBET-G							1	0.902**	0.881**	0.582*
PBET-GI								1	0.984**	0.455
SBRC-G									1	0.556*
SBRC-GI										1
Medium										
Particle size	1	-0.639*	-0.680**	-0.31	-0.938**	-0.954**	0.961**	0.861**	0.923**	0.683**
OM (mg·kg⁻¹)		1	0.591*	-0.171	0.763**	0.632*	-0.631*	-0.486	-0.545*	-0.373
Fe (mg·kg⁻¹)			1	0.312	0.814**	0.842**	-0.788**	-0.818**	-0.798**	-0.865**
pH				1	0.292	0.414	-0.382	-0.507	-0.481	-0.539*
Cr(mg·kg⁻¹)					1	0.962**	-0.943**	-0.858**	-0.913**	-0.757**
CEC (cmol⁺·kg⁻¹)						1	-0.970**	-0.934**	-0.972**	-0.846**
PBET-G							1	0.931**	0.953**	0.769**
PBET-GI								1	0.980**	0.925**
SBRC-G									1	0.897**
SBRC-GI										1
High										
Particle size	1	-0.919**	-0.632*	0.283	0.304	-0.518*	-0.897**	-0.816**	-0.800**	-0.733**

OM (mg·kg^{-1})	1	0.569*	-0.472	-0.142	0.536*	0.859**	0.784**	0.822**	0.740**
Fe (mg·kg^{-1})		1	-0.033	-0.015	0.374	0.487	0.437	0.41	0.346
pH			1	0.25	-0.149	-0.418	-0.39	-0.443	-0.387
Cr(mg·kg^{-1})				1	0.419	-0.578*	-0.596*	-0.525*	-0.588*
CEC (cmol^{+}·kg^{-1})					1	0.156	-0.024	0.022	-0.1
PBET-G						1	0.976**	0.970**	0.945**
PBET-GI							1	0.985**	0.982**
SBRC-G								1	0.989**
SBRC-GI									1

Note that "*"Significant at $p < 0.05$ level;"**"Significant at $p < 0.01$ level. "-", negative correlation; "+", positive correlation. OM: Organic Matter; CEC: Cation Exchange Capacity. SBRC: Cr bioaccessibility (%) estimated through the SBRC method; PBET: Cr bioaccessibility (%) estimated through the PBET method; "G", gastric phase; "GI", gastrointestinal phase
